# Polymer Optical Fiber Plantar Pressure Sensors: Design and Validation

**DOI:** 10.3390/s22103883

**Published:** 2022-05-20

**Authors:** Sahar Safarloo, Arántzazu Núñez-Cascajero, Ruben Sanchez-Gomez, Carmen Vázquez

**Affiliations:** 1 Electronics Technology Department, Escuela Politécnica Superior, Universidad Carlos III de Madrid, 28911 Leganés, Spain; ssafarlo@ing.uc3m.es (S.S.); arnunezc@ing.uc3m.es (A.N.-C.); 2 Nursing Department, Faculty of Nursing, Physiotherapy and Podiatry, Universidad Complutense de Madrid, 28040 Madrid, Spain; rusanc02@ucm.es

**Keywords:** fiber optic macro-bend, biofeedback tool, gait monitoring, optical fiber sensor, plastic optical fiber, plantar sensor

## Abstract

The proper measurement of plantar pressure during gait is critical for the clinical diagnosis of foot problems. Force platforms and wearable devices have been developed to study gait patterns during walking or running. However, these devices are often expensive, cumbersome, or have boundary constraints that limit the participant’s motions. Recent advancements in the quality of plastic optical fiber (POF) have made it possible to manufacture a low-cost bend sensor with a novel design for use in plantar pressure monitoring. An intensity-based POF bend sensor is not only lightweight, non-invasive, and easy to construct, but it also produces a signal that requires almost no processing. In this work, we have designed, fabricated, and characterized a novel intensity POF sensor to detect the force applied by the human foot and measure the gait pattern. The sensors were put through a series of dynamic and static tests to determine their measurement range, sensitivity, and linearity, and their response was compared to that of two different commercial force sensors, including piezo resistive sensors and a clinical force platform. The results suggest that this novel POF bend sensor can be used in a wide range of applications, given its low cost and non-invasive nature. Feedback walking monitoring for ulcer prevention or sports performance could be just one of those applications.

## 1. Introduction

Plantar pressure measurements provide significant information about foot health and functionality [1]. Areas of increased plantar pressure have been clearly related to foot pain and ulcers, especially in patients with diabetes or biomechanics foot problems [2]. Considerable progress has been made to improve plantar pressure estimation and obtain frameworks that will help comprehend general behavior or potential pathologies through investigation of the pressure created by the feet during human gait [3]. Additionally, gait pattern analysis is required to utilize automated control gadgets used for walk assistance [4].

A normal cycle of human gate is divided into stance and swing; the stance phase has 60% of total time of the cycle and refers to the moment of touching down with the floor, while swing phase refers to the air state of the lower limb (40% of the total time of the human gait). The stance phase consists of heel contact, full contact, heel off and toe off, followed by the swing phase, which includes initial swing (acceleration), mid-swing, and terminal swing (deceleration) [5]. The points with the highest localized pressure loads are observed during heel contact and toe off [6]. Plantar foot monitoring systems can be classified into three types: pressure stages, imaging innovations, and instrumented footwear measurement systems [1]. Generally, force platforms or force plates are instruments that measure the ground reaction forces generated when someone stands or jumps on it, or moves across it. They are used to quantify balance and gait. Nowadays, the study of the pressure stages has been improved, with technology that allows not only foot pressure and peak pressure to be assessed, but also temporal variables at different velocities and inclinations to be studied [7]. Even though they can offer precise estimations of 3D dynamics of the foot, they are non-portable, making them undesirable for dynamic applications, such as wearable sensors for remote sensing. Likewise, when a force plane is fixed to the ground, the patients may modify their normal stride design to place the foot within the plane limits, which brings about a wrong assessment of the patient [6,8].

The instrumented insoles were developed to relieve the force distribution platform’s limitations and to be utilized inside a shoe, bringing about a portable device that could assess the human gait outside the laboratory environment and during daily activities. Most of these insoles are based on electronic sensors, which, in some cases, lack stability and resistance to impact loads and thus introduce errors and inconsistencies [9].

To measure loading at the plantar surface, a variety of sensing technologies have been integrated to insoles, including resistive, capacitive, inductive, piezoelectric, and optical fiber sensors [10]. Resistive sensors react to mechanical deformation by varying their electrical resistance. In [11], researchers used eight fabric resistive sensors integrated with an insole to measure the plantar pressure distribution of diabetics. In [12], they developed a textile pressure sensor using a knitting technique, and a sensing matrix was integrated with a sock for pressure measurement. Resistive sensors have many advantages for plantar load sensing, including low cost, minimal interface electronics, and low sensitivity to electromagnetic interference. However, they have low repeatability [13,14], and their use in multiaxial measurements is limited. Capacitive and piezoelectric plantar pressure sensors have many hardware requirements in comparison to other techniques [15].

Optical fiber sensors exhibit various advantages in comparison with electronic sensors, such as compactness, lightweight, multiplexing capabilities, electromagnetic field immunity, electrical isolation, and biocompatibility [16]. Recent advances in biocompatible and biodegradable POF materials [17] show their potential in different biomedical applications. These features make this sensor technology ideal for measuring plantar pressure. The most common optical sensors for this application are based on fiber Bragg gratings (FBG) on silica fibers [18,19,20]. However, they have lower impact resistance, flexibility, and strain limitations compared to polymer optical fibers (POF) [21]. As an example, in [22] there is a POF-FBG sensor integrated in a cork insole to achieve higher flexibility being proposed to be integrated as part of an IoT Health solution [20]. The same technology can also be used for simultaneously measuring of the gait plantar and shear force [23]. Furthermore, POF sensors based on intensity variations are less expensive than FBG sensors in terms of the sensor itself and its interrogation system. Sensors based on intensity variation also have significant advantages in terms of implementation and signal processing. They are used for measuring temperature using macro-bends [24] or liquid level [25] as well as healthcare applications [26], including plantar pressure measurements. To avoid errors induced by undesired power fluctuations, different self-reference techniques are proposed in the literature such as those described in [27] by using wavelength division multiplexing. Speckle induced by pressing POF fibers with a piezoelectric source also allows the measuring of dynamic microvibrations [28].

There are instrumented insoles with a POF intensity sensor [29] for static and dynamic measurements of the vertical ground reaction force during gait. The sensor is based on a macro-bend POF, previously described in [30] which includes the influence of the curvature radius, lateral section length, depth, and surface roughness of POF on the sensor sensitivity, hysteresis, and linearity. The sensor has a perpendicular orientation to the insole that usually needs a special precaution to ensure the proper recovery of the fiber. For instance, in [31], the silica optical fiber loop sensor placed in the heel part of an insole, is encapsulated in silicone elastomer to ensure that the sensor reverts to its initial state after unloading. For measuring in different points, an insole with 15 intensity-based sensors multiplexed in a single POF using time-domain multiplexing methods based on side coupling between the light sources and POF lateral sections [32] as well as a multiplexed POF-embedded smart carpet for gait analysis [33] was proposed. Although multiplexing techniques can result in multipoint measurements, the requirement of using 15 light sources reduces system compactness and portability in in-shoe monitoring applications, as well as raising the total cost of the sensor system. Furthermore, due to lateral sections, these systems have high insertion losses and, as a result, higher power consumption; this effect can significantly reduce the sensitivity of sensors located further away from the detector, as mentioned in [33].

The aim of this study is to develop and verify novel macro-bend POF sensors positioned in parallel to the insole surface, not for specific clinical applications such as illness detection, but to verify its proper operation. There is no requirement for encapsulation in this approach, and the sensors can be readily steaked to any part of the insole depending on the later clinical objectives. It is an intensity POF sensor and the proposed insole consists of a single POF with two pressure sensors (with the possibility of adding more sensors) that cover high-risk points of the foot sole defined in [10]. In this system, the response acquired by one photodetector is the sum of each sensor’s contribution, rather than the individual response of each sensor, which can be acquired if the number of photodetectors is equivalent to the total number of sensors. In the first part of the experiment, a commercial force platform and a resistive sensor are used as references to be compared with the proposed POF sensor response. The second arrangement includes dynamic conditions, such as sinusoidal load tests with a universal testing machine to characterize the sensor’s ability to measure at speeds as fast as professional runners (high frequencies). Following characterization, the sensor is tested in a real-world scenario where the subject is requested to walk with the device, demonstrating the benefits of the developed insole sensor for gait analysis.

## 2. Materials and Methods

### 2.1. Sensing Element and Insoles

The POF sensor uses conventional step index HFBR-R/EXXYYYZ POF fiber which is made of polymethyl methacrylate (PMMA) with a core diameter of 980 μm, a fluorinated polymer cladding, and a polyethylene coating, resulting in a total diameter of 2 mm for the fiber considering its coating. The large diameter of this POF allows the use of low precision plastic connectors and makes cleaving and connecting easier. After cutting the fiber with a razor blade to the desired length, approximately 5 mm of the outer jacket was stripped off with a wire stripper on each side to allow the easy and proper connection of fibers into the photodiode and light source later on. The sensing element is made of loops with circular shapes and outer diameters of 1, 2, and 3 cm, named sensors 1, 2, and 3, respectively. We chose sensors with diameters equal to or greater than 1 cm, which is the critical bending radius of present POF, to prevent losing part of the optical signal due to the pre-bend. For dynamic measurements, sensor 1 is used since it has the recommended size for a plantar pressure sensor and permits a higher spatial resolution [10]. When a load is applied to the insole’s surface, a deformation occurs in the intersection part, causing the fiber to bend at this point. The transmitted optical power is attenuated because of the bending and the stress optical effect. The fiber returns to its original state when the load is removed. There are more details on the operation principle in Section 3.

The main insole utilized in this study is made of ethylene-vinyl acetate (EVA) with a thickness of 17 mm in the heel and 2 mm in the forefoot. However, we also tested the effect of insole material on sensor responsiveness using a Polypropylene (PP) insole.

### 2.2. Sensing System and Experimental Procedures

**Figure 1:**
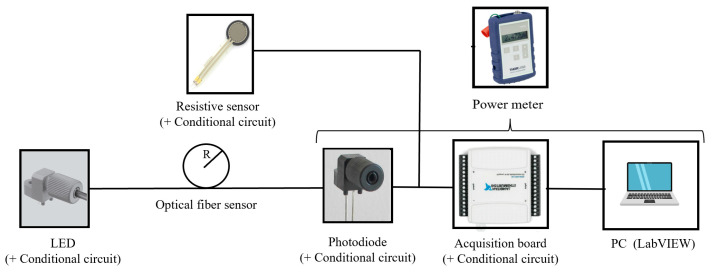
Schematic of the entire system for optical fiber sensor measurements.

For measuring purposes, the POF fiber sensors are connected to a circuit that couples light into the fiber, detects output light intensity, conditions, acquires the signal, and saves data over time. For this purpose, a LED IF-E99B with a central wavelength of 650 nm is used as a light source, and a photodiode IF-D91 is employed to acquire the power variations. The POF sensor can work with any wavelength in the transparency window of POF fiber. Finally, a DAQ National Instruments card (multi-channel data acquisition module USB-6501) acquires the sensor response and communicates with a laptop controlled by a LabVIEW program for recording the data. Figure 1 depicts a schematic of the entire system.

Tests with a commercial resistive sensor are also performed to compare the response of both sensors. The resistive sensor is also connected to the data acquisition card to perform simultaneous tests. In some parts of the experiments, a power meter is used instead of the last three elements shown in Figure 1 after the optical fiber sensor, to check the optical power value.

**Figure 2:**
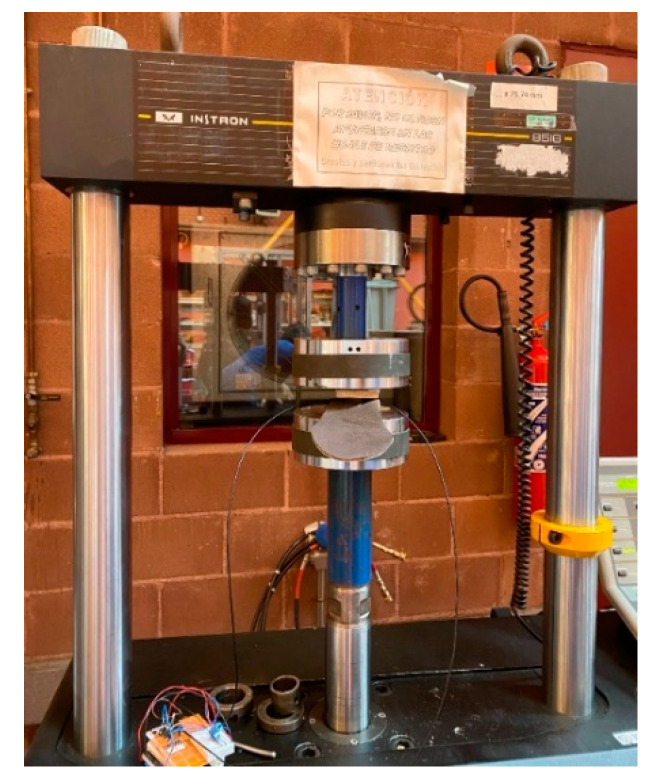
Photograph of the load testing platform with an EVA insole.

To validate the POF sensor’s response to loading and unloading, stationary and dynamic tests, checking the sensitivity and measuring range, a universal compression machine (Instron 8516) was used to perform various load tests. The data were collected at a rate of 1 kHz. Figure 2 shows a photo of the load testing platform with the EVA insole, and a piece of the same material which was placed on top of the sensor to protect it.

Using this platform, three different types of loads were applied:

- Load ramp from 0 to 2 kN, and from 2 to 0 kN with a step of 100 N (both loading and unloading).
- Sinusoidal load with an amplitude of 0.3 kN and a frequency of 0.5 Hz to simulate walking.
- Sinusoidal load with an amplitude of 0.6 kN and a frequency of 3 Hz to simulate running with the effect of impact loads.

**Figure 3:**
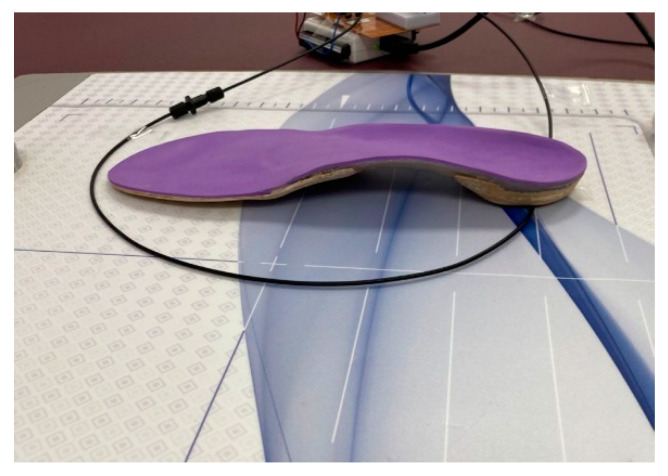
Configuration of optical sensor and insole on top of a commercial force platform.

In another part of the measurements, the results of the POF sensor were compared with a clinical pressure platform Foot print^®^. This platform has self-calibrating resistive sensors with 0.15 mm thickness and a measurement range from 0.4 to 100 N. The thickness of the force platform is 4 mm and captures 100 images/second. To accomplish the comparison, the optical fiber sensor was placed and fixed on top of the commercial force platform, and the insole was placed on the POF sensor (see Figure 3). The participant was asked to stand still on the insole, and the pressure was measured with both sensors simultaneously.

**Figure 4:**
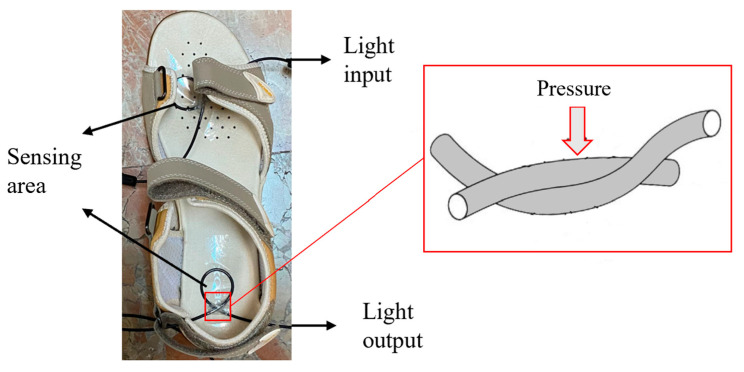
POF sensors positioned in a sandal for in-shoe measurements of plantar pressure with a zoom of intersection part of the fiber.

Finally, a dynamic test was carried out using an optical fiber sensor with two loops with a circle shape (sensing areas), one in the first metatarsal head (toe) and the other in the heel (high-risk areas). The instrumented insole was placed in a sandal for gait analysis, as shown in Figure 4, and the women participant (see next section) was asked to walk with this sandal. Figure 4 also shows the intersection of the sensing loop with a zoom of the section in which the highest bending takes place.

### 2.3. Participants

Two healthy controls (HC) participants (one female and one male; age: 26 ± 2 years; body mass index [BMI]: 26.95 ± 2.75 kg/m^2^) participated in the tests. There was no intention to derive statistical results in this research, nor to apply the sensor test to monitor a specific illness. The main purpose is to evaluate the new sensor’s capability and robustness under testing conditions, allowing future works to use patients in podiatry clinics, among others.

## 3. Results and Discussion

### 3.1. Simulations for Studying the Effect of the Bending Radius

**Figure 5:**
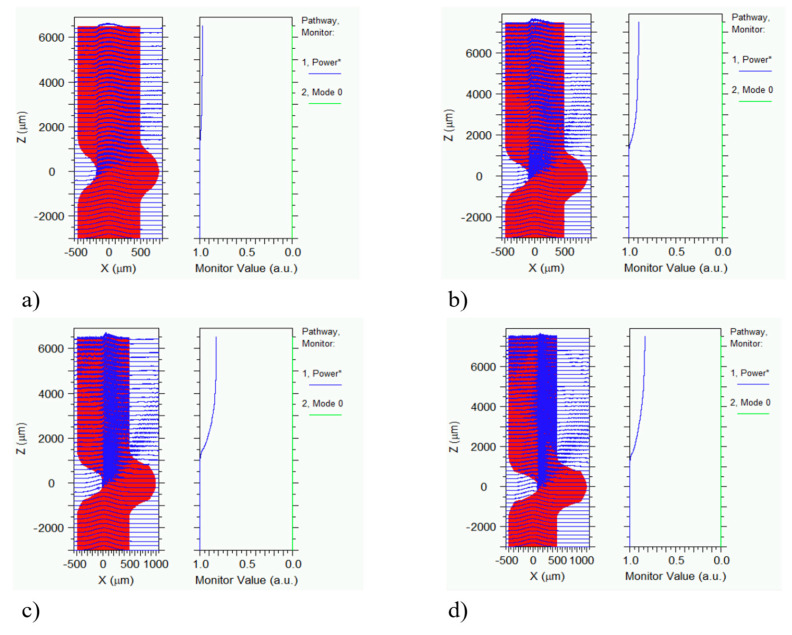
The transmitted optical power with varying bending diameters simulated by Beam-PROP: (**a**) 3.9 mm, (**b**) 3 mm, (**c**) 2.5 mm, (**d**) 2 mm.

BeamPROP from RSoft was used to simulate the effect of bending at the intersection of fiber loops, which is clearly shown in Figure 4. Bending in this area plays a vital role in the sensor’s response, and the bending diameter can range from 2 mm (smallest value), when the upper fiber is totally pushed forward the lower fiber, to infinite, when the fiber is under no force. The results in Figure 5 illustrate light propagation along the bent optical fiber with total power values. Figure 5b represents the limit case previously described. According to these results, for a bending diameter of 3.9 mm, 98% of the light will be transmitted through this section of fiber. However, as we reduce the bending diameter, the amount of transmitted power will decrease as well. It was also discovered that the output intensity versus applied curve radius exhibits a slight nonlinearity, which agrees with the study results of [34].

In addition to the bending loss, there is also attenuation related to the stress optical effect. When stress is applied to a fiber, an amount of anisotropy will be produced in its optical properties. In this case, the refractive index change will be equal to [35]:

Δn_z_ = −*n*_*co*_^3^*q*_11_*xE*/2*R* (1)

where *n_co_* is the refractive index of the core before the applied stress, *E* is Young’s modulus, *R* is the bending radius, *q*_11_ is the stress optical coefficient of the material, and z is the direction of applying load. This change in the refractive index can be a source of loss in the fiber. Therefore, we repeated the simulations with the stress-induced changes in the refractive index of the fiber based on (1), and we realized that the optical loss due to this effect was negligible and the main effect, in this case, is related to the bending conditions previously described.

### 3.2. Validation of POF Response with a Commercial Piezoresistive Sensor

**Figure 6:**
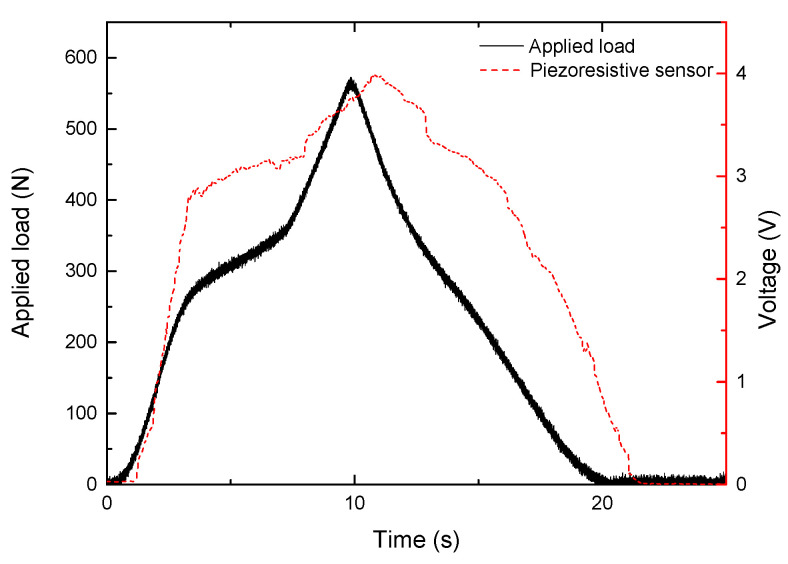
Applied load shape on the resistive sensor and response of the piezoresistive sensor to the applied load.

First, the behavior of a commercial piezoresistive sensor was investigated by applying a load and unload ramp from 0 to 500 N in steps of 100 N using the compression machine. Figure 6 illustrates the graph of applied load and the sensor’s response. According to these results, the resistive sensor can detect the overall shape of the applied load; nevertheless, this sensor lacks good repeatability, as the response varied by up to ±1.5 V when we repeated the test. Furthermore, according to the datasheet, this resistive sensor can only detect applied weights up to 50 kg (approximately 500 N).

**Figure 7:**
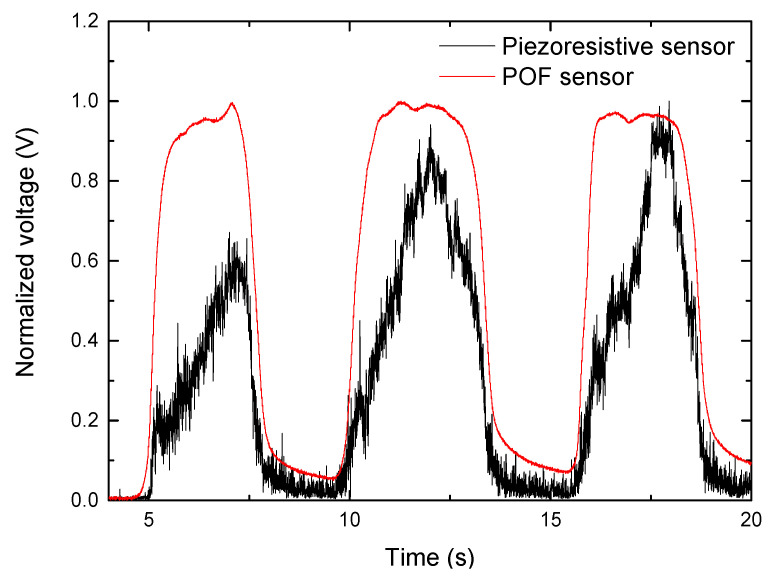
Simultaneous response of POF and piezoresistive sensors at the heel part of the insole.

Then, simultaneous tests with the piezoresistive sensor were carried out to validate the POF sensor. Both sensors were placed in the insole’s heel section, and their responses were compared. Three repetitions were performed during the measurements, with the woman participant standing on the insole for about 4 s and then moving back for 2 s. Figure 7 presents the results for both sensors and it shows that the loading and unloading points are in the same positions in the time axis; however, the POF sensor exhibits higher stability and repeatability, as well as less noise in the response. This could also validate the POF sensor’s response value, as the maximum normalized voltage levels in the second and third tests are nearly identical in both sensors. Furthermore, the POF sensor initial condition is not fully recovered after 2 s as a residual voltage is present after unloading.

### 3.3. Validation of POF Response with a Commercial Force Platform

In this section, we compare the results obtained by the POF sensor and a commercial clinical force platform at the same time. The measurements in two different parts of the insole, as well as the use of different insole materials, will be discussed in the following sections.

#### 3.3.1. Results on Different Parts of the Insole

**Figure 8:**
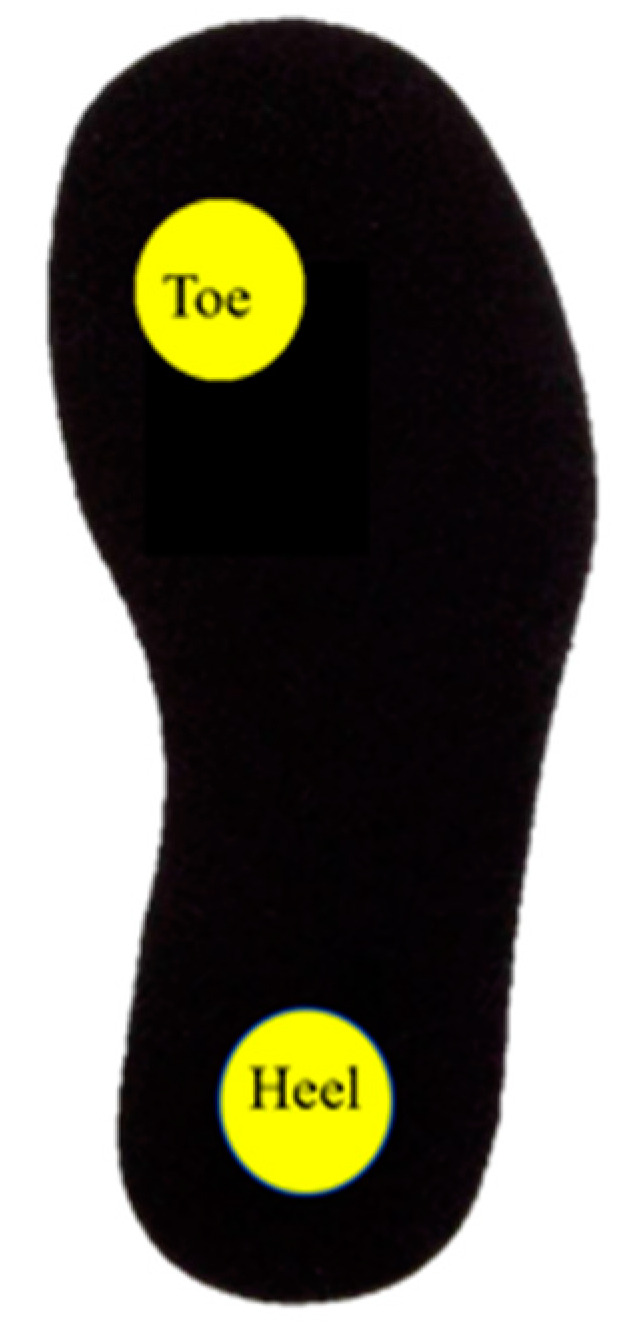
The position of the sensors on the heel and toe part of the insole which are related to the point with higher pressure in the sole of the foot.

The individual POF sensor was first located at the center of the heel part and then in the toe part (see Figure 8) to measure the pressure at each part of the insole separately. When the woman participant was standing on the insole, the pressure with the force platform and the voltage change with the POF sensor setup was captured at the same time. Each test was repeated three times and the results are shown in Table 1.

If we compare both zones, it is shown that both sensors achieve lower values in the toe part than in the heel part as was expected. The pressure in the toe part of the POF response is reduced by 55.9%, and the pressure in the toe part of the force platform is reduced by 57.8% in comparison with the heel part. Therefore, the POF sensor is detecting the changes in pressure with an error less than 3% and the normalized response can be used to monitor the plantar pressure.

#### 3.3.2. Effect of Different Insole Materials

As the different parts of the insole or even different insoles can be made of different materials, we have tested the POF sensor with two types of materials specifically used for this purpose by the podiatrists. In our case, the POF sensor is located at the heel part in two different insoles, one made of ethylene-vinyl acetate (EVA), and the other of polypropylene (PP). All the steps were completed using the same procedure as in the previous section, with the results provided in Table 2. Both sensors detect a lower pressure when EVA is used as insole material since PP is a more rigid material.

According to the data obtained by the POF sensor, the pressure with the EVA is equal to 59.6% of the pressure with PP. This value is equal to 64.9%, according to the results of the commercial sensor. Therefore, the POF sensor is detecting the same track that the commercial force platform can detect.

### 3.4. Validation and Calibration of POF Response with Load Tests

**Figure 9:**
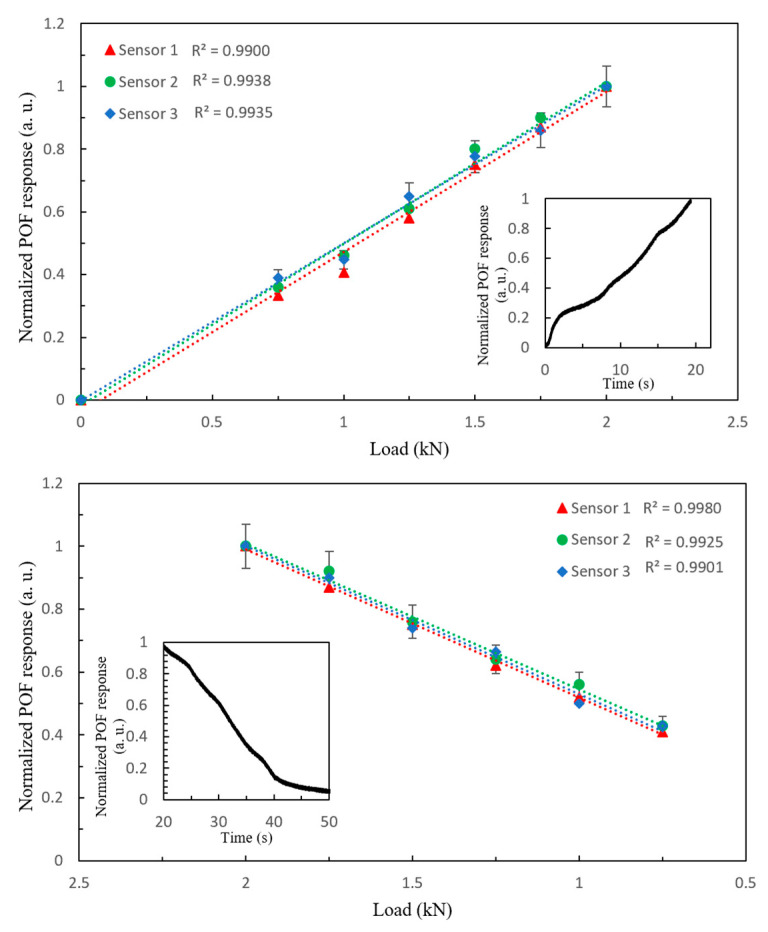
Applied load and normalized POF sensor response for a load and unload ramp of 2 kN with 10 N step for three different sensors.

The POF sensors were calibrated with the compression machine using different load tests. In the first experiment, we ramped up from 0 to 2 kN with a step of 100 N, and then ramped down to the initial position. The measurements were repeated three times for each sensor. Figure 9 depicts the applied load and the corresponding normalized POF sensor response as a function of applied load for the three different sensors described in 2.1. For the sake of clarity, the error bars are only shown for one of the sensors. According to these results, the fiber response resembles the applied load and is linear over this large pressure range since the correlation coefficient (R^2^) between sensor responses and linear regression is greater than 0.99 in all cases, which is consistent with the result reported in [32]. According to the literature, the maximum pressure exerted on heels as a percentage of body weight (BW) can range from 2.5% BW/cm^2^ for young people to 4.5% BW/cm^2^ for the elderly [36]. These numbers are based on measurements taken in a dynamic environment. As a result, during a walk, this sensor can measure the pressure at the heel part for an elderly person with a body mass of 400 kg.

It has to be taken into account that the response of the POF sensor is not able to completely recover the steady state after the measurement; there is a small increase in the signal, as previously shown, however this increase does not affect the linearity of the sensors. Despite the higher optical losses in sensors 2 and 3 due to their larger area exposed to pressure, the normalized sensitivity of all three sensors is 0.507 ± 0.03 kN^−1^, which is one order of magnitude smaller than the sensitivity of POF sensors based on intensity variation in lateral section reported in [29,32] but with a simpler configuration. According to [30], the lateral section parameters and the sensor’s radius of curvature, which is related to the sensor mounting, affect the sensitivity of intensity variation sensors based on lateral section. However, in this study, the sensitive zone is the fiber intersection part, which has the same characteristics for all three sensors, so not only is there no need to include the lateral section, but the sensors’ sensitivities remain constant even when the curvature (diameter of loop in this case) changes. Moreover, removing the jacket and a part of the cladding reduces fiber protection and increases optical losses, both of which can result in a smaller measurement range. Table 3 briefly summarizes the previous studies based on optical fiber sensors.

To further verify the operation of the sensors, both participants were asked to stand still on sensor 1 with heel part of their sole for about 5 s to record the data. Considering the normalization for these two pressure points, the calculated sensitivity is equal to 0.651 kN^−1^, which is higher than the sensitivity reported in the previous paragraphs. This difference might be due to the errors of foot positioning. Moreover, the pressure at heel part of both young participants is supposed to be 2.5% BW; however, the pressure distribution varies between different people [36]. Nevertheless, the weight of each voluntary has no effect on the gait analysis, since the normalized sensor response is used for each weight, and the aim of this work is a qualitative analysis.

**Figure 10:**
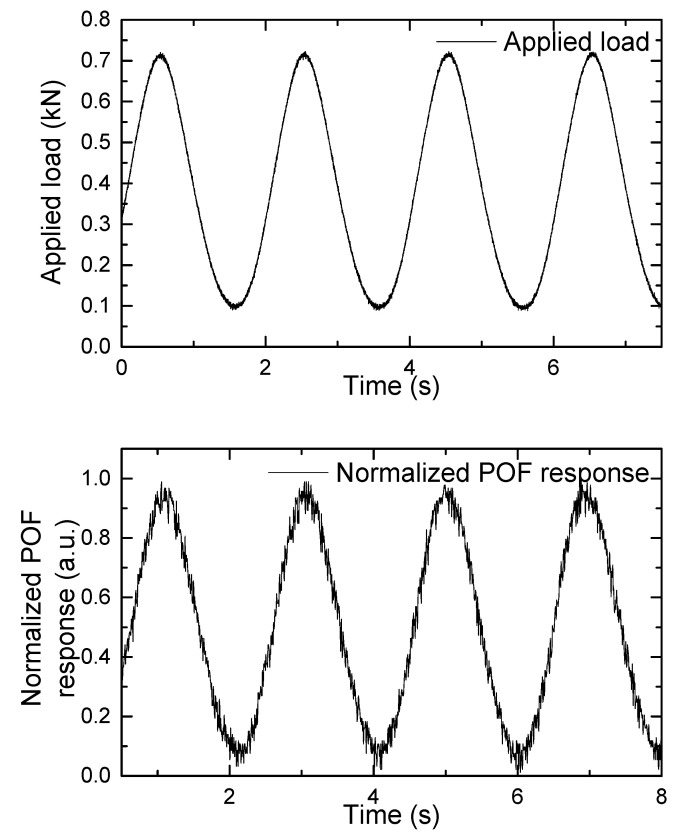
Applied load and normalized POF response that simulate a walking pattern.

**Figure 11:**
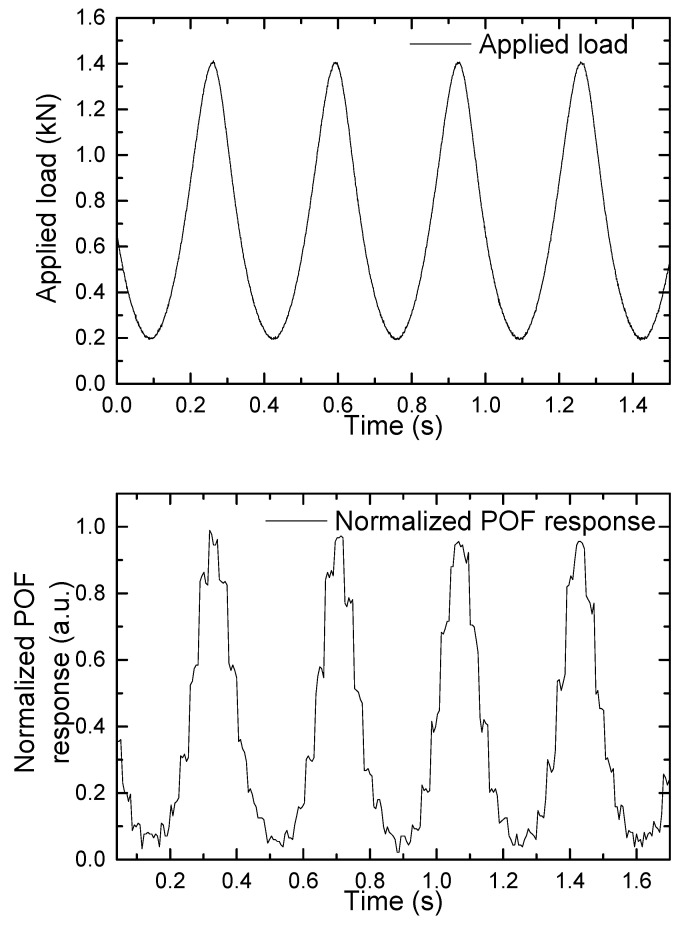
Applied load and normalized POF response that simulate a running pattern.

Afterward, cyclic tests were carried out in order to simulate walking and running patterns while minimizing the effects of temperature changes, speed inconsistency, posture changes, and other factors that occur during human gait. Figure 10 and Figure 11 show the applied load and associated normalized POF response that simulates walking and running patterns, respectively.

These results demonstrated the POF sensor’s repeatability as well as its ability to take measurements at frequencies as high as a champion athlete’s running frequency. To the best of our knowledge, this is the first time such a measurement has been reported.

Improvements in the acquisition system in the POF sensor should be applied in the future, increasing the number of bits, to avoid limitations in the resolution of the measurements. It is also appreciated that there is some delay in the system response that needs further analysis.

### 3.5. Dynamic Analysis with Participants

**Figure 12:**
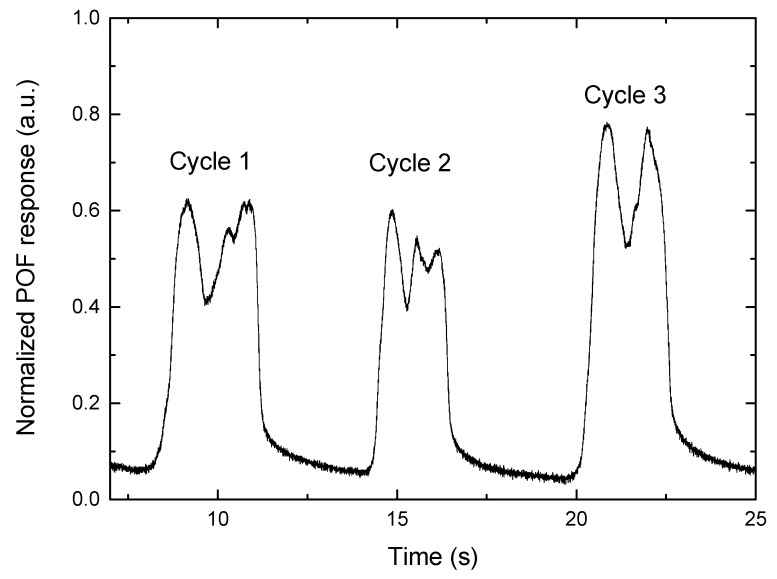
In-shoe monitoring of sequential gait cycles.

After the insole characterization and the comparison with commercially available sensors, the last test is the evaluation of the proposed instrumented insole with POF sensors on gait analysis. A dynamic test using an optical fiber sensor with two sensing areas, one located at the toe and the other at the heel, was carried out. For this purpose, the plastic optical fiber sensors were placed in a sandal’s insole, as shown in Figure 4, once the sensors were located, the woman participant walked with it, to monitor the gait. The normalized results for three cycles are shown in Figure 12. The M-shape is shown for the plantar pressure, which is a common pattern that has been studied extensively over the years [38]. At the end of the stance phase, when the ground reaction force is zero, the swing phase begins. These results demonstrate the system’s capability for gait analysis. Furthermore, all of the stance phase’s divisions can be separated, although because of the dynamic character of a gait cycle, some stages can be represented as intervals. When the heel reaches the ground, the heel strike phase begins.

When the first peak of the load is measured, the maximum weight acceptance phase is reached. The foot then begins to rotate with the ankle acting as a pivot until it reaches the full contact phase when the foot is straight, and the heel off phase occurs when the heel loses contact with the ground. Because the entire body weight is transmitted to the metatarsal region, there is another peak of load at this location (second peak at each cycle). When the foot loses touch with the ground, the last phase, toe off, occurs and the swing phase begins [29]. As a result, the plantar pressure distribution at each gait phase is consistent with the widely reported plantar pressure distribution [38]. This feature, combined with the validation on a force platform and resistive sensors, indicates that the proposed POF sensor is capable of measuring plantar pressure in a variety of scenarios (static and dynamic tests). Finally, there is some inherent variability in gait in the same participant with different speeds and gestures, so the repeatability of the gait needs to be further investigated in future works. The simulating gait with sinusoidal loads in Section 3.4 showed that the load pattern is consistent (with smaller errors than the human gait), allowing a proper calibration of the sensor’s response.

## 4. Conclusions

The proposed plastic optical fiber macro-bend in this study has high robustness and portability, as well as proven reliability for both static and dynamic measurements, making it promising for a wide range of biofeedback applications. The proposed sensor was tested over the widest measuring force range (0–2 kN) as well as highest frequency (3 Hz) not previously reported in the literature with a novel method that enables the use of the sensor for subjects with obesity as well as runners. It also shows high versatility as it is possible to use it in many types of shoes in a straightforward way which is interesting for real-world applications. Thanks to its portability, the sensor might be utilized to track walking without restricting the subject’s movements. The sensor also has the advantage of being simple to produce and inexpensive. Special optical fibers, encapsulant materials, or several light sources and photodetectors are not required. Furthermore, no specific signal processing is required for sensor interrogation, and when compared to commercial pressure platforms, it gives similar results.

The multiplexing capability of this sensor can be tested in future works by using a splitter and increasing the number of photodetectors to the desired number of sensors to obtain the response of each sensor individually.

## Figures and Tables

**Table 1:** Response of the force platform and POF sensor at heel and toe.

Sensor Type	Average Response in the Heel	Average Response in the Toe
POF	0.34 (V)	0.15 (V)
Force platform	758 (gr/cm^2^)	320 (gr/cm^2^)

**Table 2:** Response of the force platform and POF with different insole materials at the heel.

Material/Sensor Type	Response of POF	Response of Force Platform
EVA	0.34 (V)	758 (gr/cm^2^)
PP	0.57 (V)	1168 (gr/cm^2^)
(EVA/PP) × 100	59.6%	64.9%

**Table 3:** Summary of optical fiber sensors’ characteristics.

Fiber *	Principle	Sensitivity	Shape	Measurment Range	Linearity	Losses/ Complexity	Multiplexing	Ref.
POF	Intensity variation	1.03–97.66 N^−1^ **	Curvature with lateral section	0–50 N ***	_	High/High	Yes	[33]
POF	Intensity variation	_	Curvature with lateral section	0–50 N 46–97 kg	R^2^ > 0.99	High/High	Yes	[32]
LPS-POF	Intensity variation	143.21 ADC/kPa	Straight	1–4 N		Low/High	No	[37]
POF	Intensity variation	0.0086–0.0089 N^−1^	Curvature with lateral section	0–50 N 54–98 kg		High/High	No	[29]
SOF	Intensity variation	0.16–0.31 N^−1^ **	Circular loop	0–80 N	Non-linear	Low/High	No	[31]
POFBG	FBG	8 pm/KPa	Straight	0–118 N	0.982 < R^2^ < 0.994	Low/High	No	[22]
POF	Intensity variation	0.00051 N^−1^	Horizontal curvature	0–2kN	R^2^ > 0.99	Low/Low	No	This work

* POF (Polymer Optical Fiber), LPS-POF (Light Polymerization Spinning POF), SOF (Silica Optical Fiber), and POFBG (Polymer Optical Fiber Bragg Gratings). ** Sensitivity is dependant on positioning and is calculated without normalization. *** Tendency to saturation for higher weights.

## Data Availability

The Data Protection Delegate from Universidad Carlos III Madrid emitted a positive report on 9 May 2022 pointing out that the research activity is conducted in accordance with the regulations for the protection of data.

## References

[1] Ramirez-Bautista J.A., Huerta-Ruelas J.A., Chaparro-Cárdenas S.L., Hernández-Zavala A. (2017). A Review in Detection and Monitoring Gait Disorders Using In-Shoe Plantar Measurement Systems. IEEE Rev. Biomed. Eng..

[2] Bowers A.L., Castro M.D. (2007). The mechanics behind the image: Foot and ankle pathology associated with gastrocnemius contracture. Semin. Musculoskelet. Radiol..

[3] Wafai L., Zayegh A., Woulfe J., Mahfuzul S., Begg R. (2015). Identification of foot pathologies based on plantar pressure asymmetry. Sensors.

[4] Villa-Parra A.C., Delisle-Rodriguez D., Lima J.S., Frizera-Neto A., Bastos T. (2017). Knee impedance modulation to control an active orthosis using insole sensors. Sensors.

[5] Oladeji O., Stackhouse C., Gracely E., Orlin M. (2008). Comparison of the two-step and midgait methods of plantar pressure measurement in children. J. Am. Pod. Med. Assoc..

[6] Abdul Razak A.H., Zayegh A., Begg R.K., Wahab Y. (2012). Foot plantar pressure measurement system: A review. Sensors.

[7] Zulkifli S.S., Loh W.P. (2020). A state-of-the-art review of foot pressure. Foot Ankle Surg..

[8] Ballaz L., Raison M., Detrembleur C. (2013). Decomposition of the vertical ground reaction forces during gait on a single force plate. J. Musculoskelet. Neuronal Interact..

[9] Leal-Junior A.G., Domingues M.F., Min R., Vilarinho D., Theodosiou A., Tavares C., Alberto N., Leitão C., Kalli K., Frizera-Neto A. (2019). Fiber Bragg Based Sensors for Foot Plantar Pressure Analysis. Commun. Comput. Inf. Sci..

[10] Wang L., Jones D., Chapman G.J., Siddle H.J., Russell D.A., Alazmani A., Culmer P. (2020). A Review of Wearable Sensor Systems to Monitor Plantar Loading in the Assessment of Diabetic Foot Ulcers. IEEE Trans. Biomed. Eng..

[11] Shu L., Mai K.Y., Tao X.M., Li Y., Wong W.C., Lee K.F., Yip S.L., Shum W.H.A., Chan W.L., Yuen C.P. Monitoring diabetic patients by novel intelligent footwear system. Proceedings of the 2012 International Conference on Computerized Healthcare (ICCH).

[12] Lin X., Seet B.C. (2017). Battery-Free Smart Sock for Abnormal Relative Plantar Pressure Monitoring. IEEE Trans. Biomed. Circuits Syst..

[13] Giovanelli D., Farella E. (2016). Force Sensing Resistor and Evaluation of Technology for Wearable Body Pressure Sensing. J. Sensors.

[14] Del Prete Z., Monteleone L., Steindler R. (2001). A novel pressure array sensor based on contact resistance variation: Metrological properties. Rev. Sci. Instrum..

[15] Tsai T.M., Tsou C., Huang P.W., Lee S.Y., Chang S.J. (2020). Monitoring System with Cross-Type Capacitive Plantar Pressure Sensor. IEEE Sens. J..

[16] Webb D.J. (2015). Fibre Bragg grating sensors in polymer optical fibres. Meas. Sci. Technol..

[17] Wang Y., Huang Y., Bai H., Wang G., Hu X., Kumar S., Min R. (2021). Biocompatible and biodegradable polymer optical fiber for biomedical application: A review. Biosensors.

[18] Liang T.C., Lin J.J., Guo L.Y. (2016). Plantar pressure detection with fiber bragg gratings sensing system. Sensors.

[19] Suresh R., Bhalla S., Hao J., Singh C. (2015). Development of a high resolution plantar pressure monitoring pad based on fiber Bragg grating (FBG) sensors. Technol. Heal. Care.

[20] Domingues M.F., Alberto N., Leitao C.S.J., Tavares C., De Lima E.R., Radwan A., Sucasas V., Rodriguez J., Andre P.S.B., Antunes P.F.C. (2019). Insole Optical Fiber Sensor Architecture for Remote Gait Analysis—An e-Health Solution. IEEE Internet Things J..

[21] Peters K. (2011). Polymer optical fiber sensors—A review. Smart Mater. Struct..

[22] Vilarinho D., Theodosiou A., Leitão C., Leal-Junior A.G., de Fátima Domingues M., Kalli K., André P., Antunes P., Marques C. (2017). POFBG-embedded cork insole for plantar pressure monitoring. Sensors.

[23] Tavares C., Leite F., Domingues M.D.F., Paixao T., Alberto N., Ramos A., Silva H., Antunes P.F.D.C. (2021). Optically Instrumented Insole for Gait Plantar and Shear Force Monitoring. IEEE Access.

[24] Moraleda A.T., Vázquez C., Zaballa J.Z., Arrue J. (2013). Temperature sensor based on a polymer optical fiber macro-bend. Sensors.

[25] Montero D.S., Lallana P.C., Vázquez C. (2012). A polymer optical fiber fuel level sensor: Application to paramotoring and powered paragliding. Sensors.

[26] Leal-Junior A.G., Diaz C.A.R., Avellar L.M., Pontes M.J., Marques C., Frizera A. (2019). Polymer optical fiber sensors in healthcare applications: A comprehensive review. Sensors.

[27] Tapetado A., Pinzón P.J., Zubia J., Vázquez C. (2015). Polymer Optical Fiber Temperature Sensor With Dual-Wavelength Compensation of Power Fluctuations. J. Light. Technol..

[28] Pinzon P.J., Montero D.S., Tapetado A., Vazquez C. (2017). Dual-wavelength speckle-based SI-POF sensor for cost-effective detection of microvibrations. IEEE J. Sel. Top. Quantum Electron..

[29] Leal-Junior A.G., Frizera A., Avellar L.M., Marques C., Pontes M.J. (2018). Polymer Optical Fiber for In-Shoe Monitoring of Ground Reaction Forces during the Gait. IEEE Sens. J..

[30] Leal-Junior A.G., Frizera A., José Pontes M. (2018). Sensitive zone parameters and curvature radius evaluation for polymer optical fiber curvature sensors. Opt. Laser Technol..

[31] Kamizi M.A., Negri L.H., Fabris J.L., Muller M. (2019). A smartphone based fiber sensor for recognizing walking patterns. IEEE Sens. J..

[32] Leal-Junior A.G., Díaz C.R., Marques C., Pontes M.J., Frizera A. (2019). 3D-printed POF insole: Development and applications of a low-cost, highly customizable device for plantar pressure and ground reaction forces monitoring. Opt. Laser Technol..

[33] Avellar L.M., Leal-Junior A.G., Diaz C.A.R., Marques C., Frizera A. (2019). POF smart carpet: A multiplexed polymer optical fiber-embedded smart carpet for gait analysis. Sensors.

[34] Ledoux W.R., Wang W.C. (2007). Composite optical bend loss sensor for pressure and shear measurement. IEEE Sens. J..

[35] Zubia J., Arrue J., Mendioroz A. (1997). Theoretical Analysis of the Torsion-Induced Optical Effect in a Plastic Optical Fiber. Opt. Fiber Technol..

[36] Hessert M.J., Vyas M., Leach J., Hu K., Lipsitz L.A., Novak V. (2005). Foot pressure distribution during walking in young and old adults. BMC Geriatr..

[37] Leal-Junior A., Campos V., Frizera A., Marques C. (2020). Low-cost and high-resolution pressure sensors using highly stretchable polymer optical fibers. Mater. Lett..

[38] Kirtley C. (2006). Clinical Gait Analysis: Theory and Practice.

